# Supplementary material for: Reduced mGluR5 Activity Modulates Mitochondrial Function
Source: Cells. 2021 Jun 2;10(6):1375. doi: 10.3390/cells10061375 (PMC8228325; doi:10.3390/cells10061375)
Supplement: Supplementary file 1 [file cells-10-01375-s001.zip › Gonzalez-Lozano_mGluR5_SupplementaryFigures.pdf]

Supplementary Data

Data S1. Complete results table of the mass spectrometry analysis of mGluR5 KO synaptosomes. (**A**) Quantitative and statistical analysis of all proteins identified with at least two high quality peptides in WT and mGluR5 KO synaptosomes. Mitochondrial proteins were labeled as retrieved from MitoMinner. (**B**) Gene ontology enrichment analysis for the significantly regulated proteins (eBayes $p < 0.01$) in mGluR5 KO. Proteins with increased (unregulated) and decreased (downregulated) expression in mGluR5KO were analyzed separately

Data S2. Complete results table of the mass spectrometry analysis of CTEP-treated mice. (**A**) Quantitative and statistical analysis of all proteins identified with at least two high quality peptides in synaptosomes of CTEP-treated mice scarified 2 (CTEP2h), 24 (CTEP1d) and 48 hours (CTEP2d) after the first administration. Mitochondrial proteins were labeled as retrieved from MitoMinner. (**B**) Gene ontology enrichment analysis for the significantly regulated proteins (eBayes $p < 0.01$) in CTEP2h group compared to vehicle control. Proteins with increased (unregulated) and decreased (downregulated) expression in CTEP-treated mice were analyzed separately

Data S3. Cell-type enrichment analysis table of CTEP-induced mitochondrial protein expression regulation. (**A**) Complete results of expression weighted cell-type enrichment (EWCE) analysis on the differentially abundant mitochondrial proteins and the 50 mitochondrial proteins with larger fold-changes upon CTEP2h treatment. (**B**) Extended cell-type enrichment analysis (FUMA) of the differentially abundant mitochondrial proteins upon CTEP-treatment (CTEP2h group) using 29 different scRNA-seq datasets from mouse and human nervous system tissue

Supplementary Figures

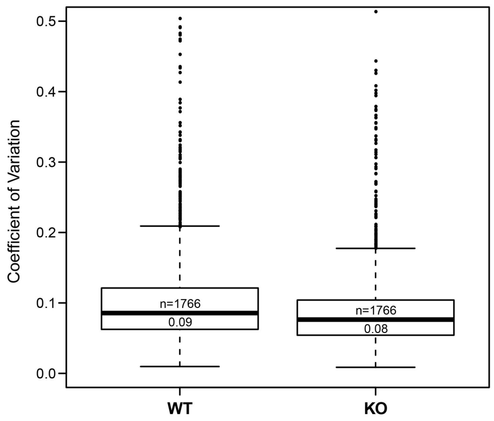

Figure S1. Quality control for the quantitative proteomics analysis of mGluR5 KO synaptosomes. Boxplot showing the distribution of coefficients of variation for all quantified proteins in WT and mGluR5 KO synaptosomes (N=6)

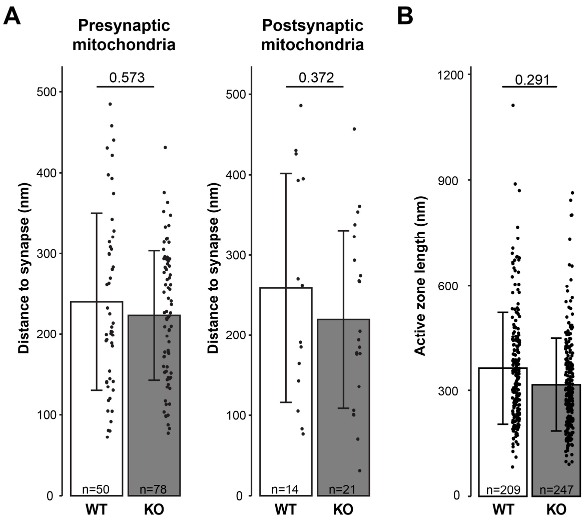

Figure S2. Electron microscopy analysis of mGluR5 KO synaptic mitochondria. (**A**) Linear distance from the presynaptic and postsynaptic mitochondria to the active zone and postsynaptic density, respectively. No significant difference was found between WT and KO. (B) Active zone length for WT and KO synapses (N=6, n=209 for WT; N=6, n=247 for KO). All bar graphs, means ± SD

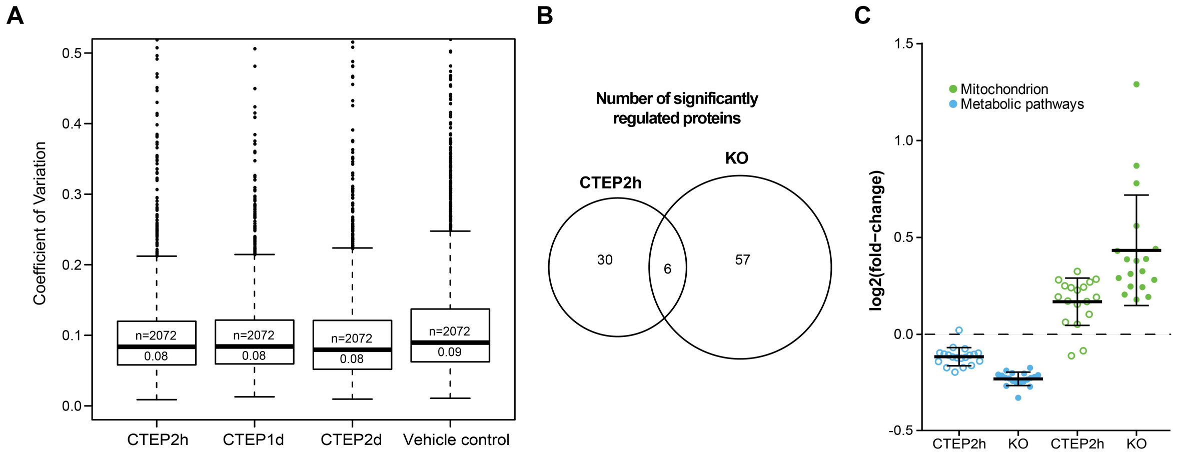

Figure S3. Quality control for the quantitative proteomics analysis of CTEP-treated mice synaptosomes and comparison with mGluR5 KO dataset. (**A**) Boxplot showing the distribution of coefficients of variation for all quantified proteins in synaptosomes from the different CTEP-treatment groups and vehicle control (N=6). (**B**) Venn diagram showing the number of significantly regulated proteins identified in both CTEP2h and mGluR5 KO proteomic datasets. (**C**) Expression fold-changes in CTEP2h and mGluR5 KO datasets for the significantly regulated proteins in mGluR5 KOs annotated as “mitochondrion” and “metabolic pathways” by enrichment analysis (Data S2B). Scatter plot, means ± SD

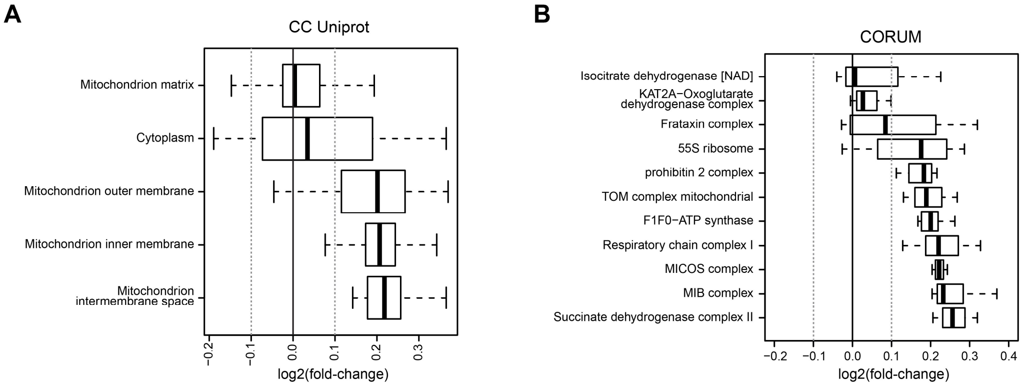

Figure S4. Characterization of CTEP-induced mitochondrial protein expression regulation. (**A**) Boxplot showing the distribution of mitochondrial protein expression fold-changes (CTEP2h group) in different cellular components as retrieved from Uniprot. Solid and dotted vertical lines indicate no change and ±0.1 fold-change (log2), respectively. (**B**) Boxplot showing the distribution of mitochondrial protein expression fold-changes (CTEP2h group) in different protein complexes based on CORUM database. Solid and dotted vertical lines indicate no change and ±0.1 fold-change (log2), respectively

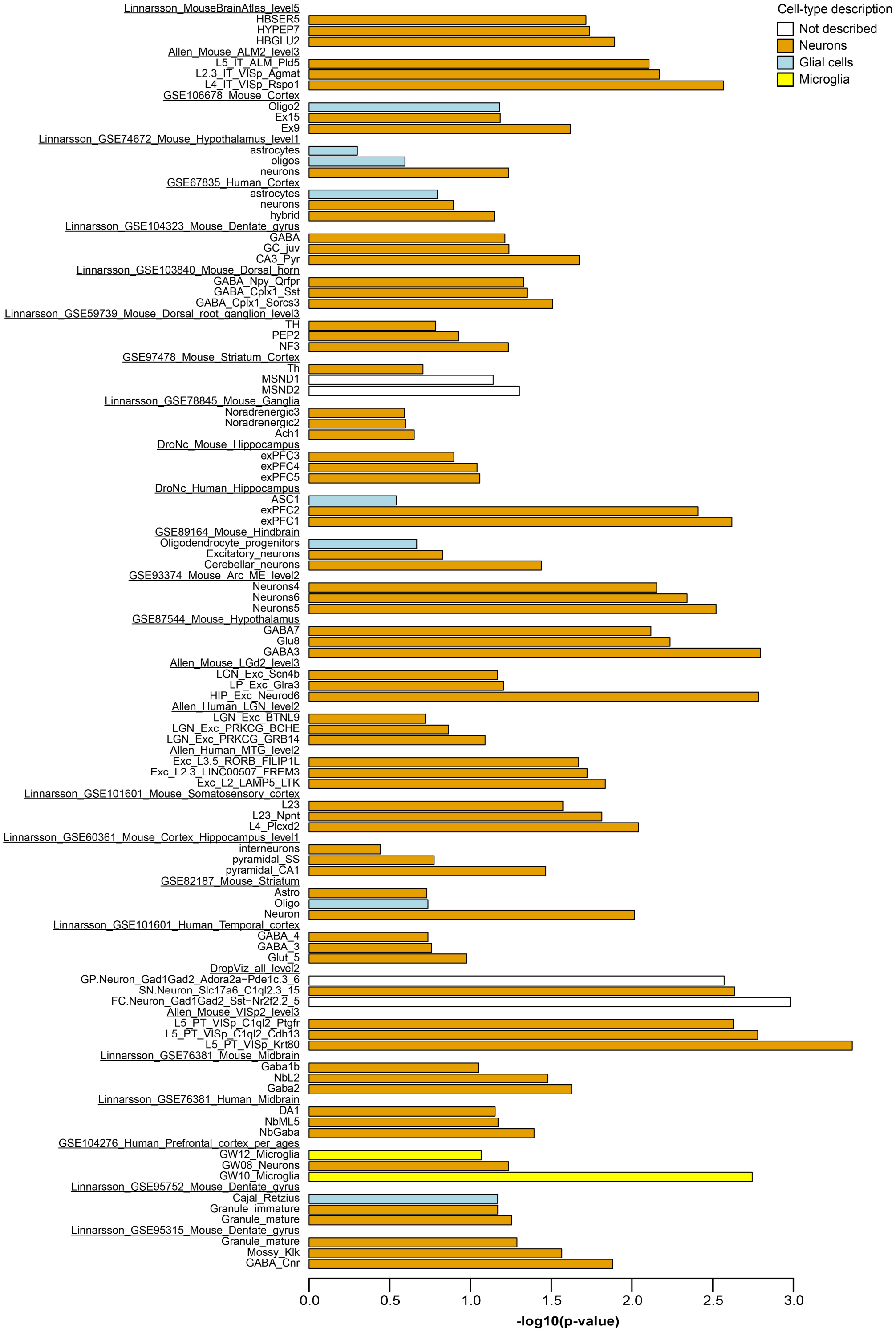

Figure S5. Cell-type enrichment analysis of CTEP-induced mitochondrial protein expression regulation. Cell-type enrichment analysis of the differentially abundant mitochondrial proteins upon CTEP-treatment (CTEP2h group). The three cell-types with lower p-value are depicted for 29 different scRNA-seq datasets from mouse and human nervous system tissue as implemented in FUMA (Data S3B). Colors indicate the general cell-type category

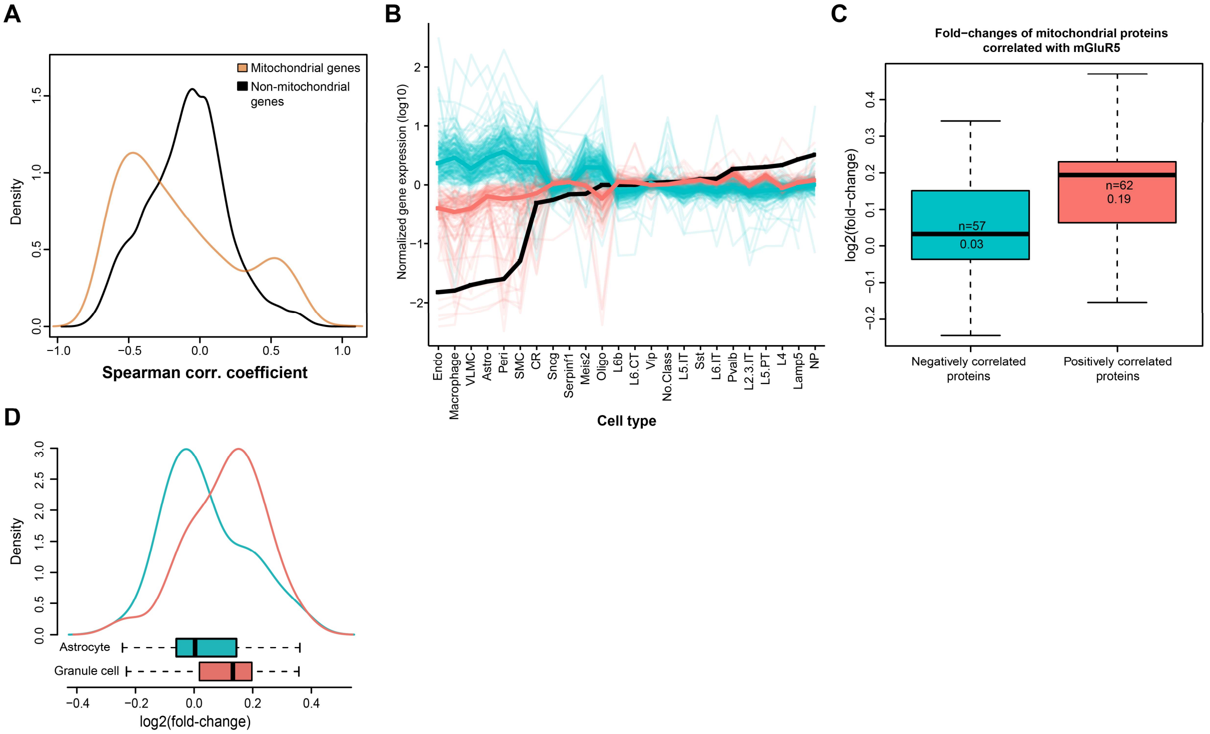

Figure S6. Gene expression correlation analysis between mGluR5 and mitochondrial genes. (**A**) Distribution of Spearman correlation coefficients from the gene expression correlation of all mitochondrial and non-mitochondrial genes with mGluR5. Positive, negative and cero correlation coefficient values indicate gene expression correlation, anti-correlation and no correlation, respectively. (**B**) Normalized gene expression across the different cell-types for the mitochondrial genes significantly correlated with mGluR5 (FDR corrected $p < 0.05$). Red and blue lines indicate genes positively and negatively correlated with mGluR5, respectively. Thick lines represent the median expression of the individual genes in each category. mGluR5 gene expression is depicted in black as reference. Positive and negative correlations correspond mainly to genes little and highly expressed in non-neuronal cell types, respectively. (**C**) Boxplot showing the distribution of protein expression fold-changes upon CTEP-treatment (CTEP2h group) for the mitochondrial proteins positively and negatively correlated with mGluR5. Colors match the gene categories in panel **B**. (**D**) Boxplot and distribution of protein expression fold-changes induced by the treatment with CTEP (CTEP2h group) for granule cells and astrocytes mitochondrial marker proteins, as determined by single-cell protein expression (Fecher *et al.*, 2019). Granule cell and astrocyte markers are indicated in red and blue, respectively.
